# Mathematical model predicts response to chemotherapy in advanced non-resectable non-small cell lung cancer patients treated with platinum-based doublet

**DOI:** 10.1371/journal.pcbi.1008234

**Published:** 2020-10-05

**Authors:** Emilia Kozłowska, Rafał Suwiński, Monika Giglok, Andrzej Świerniak, Marek Kimmel

**Affiliations:** 1 Department of Systems Biology and Engineering, Silesian University of Technology, Akademicka Gliwice, Poland; 2 The 2^nd^ Radiotherapy and Chemotherapy Clinic, M. Sklodowska-Curie National Research Institute of Oncology, Gliwice Branch, Gliwice, Poland; 3 Departments of Statistics and Bioengineering, Rice University, Houston Texas, United States of America; King’s College London, UNITED KINGDOM

## Abstract

We developed a computational platform including machine learning and a mechanistic mathematical model to find the optimal protocol for administration of platinum-doublet chemotherapy in a palliative setting. The platform has been applied to advanced metastatic non-small cell lung cancer (NSCLC). The 42 NSCLC patients treated with palliative intent at Maria Sklodowska-Curie National Research Institute of Oncology, Gliwice Branch, were collected from a retrospective cohort of patients diagnosed in 2004–2014. Patients were followed-up, for three years. Clinical data collected include complete information about the clinical course of the patients including treatment schedule, response according to RECIST classification, and survival. The core of the platform is the mathematical model, in the form of a system of ordinary differential equations, describing dynamics of platinum-sensitive and platinum-resistant cancer cells and interactions reflecting competition for space and resources. The model is simulated stochastically by sampling the parameter values from a joint probability distribution function. The machine learning model is applied to calibrate the mathematical model and to fit it to the overall survival curve. The model simulations faithfully reproduce the clinical cohort at three levels long-term response (OS), the initial response (according to RECIST criteria), and the relationship between the number of chemotherapy cycles and time between two consecutive chemotherapy cycles. In addition, we investigated the relationship between initial and long-term response. We showed that those two variables do not correlate which means that we cannot predict patient survival solely based on the initial response. We also tested several chemotherapy schedules to find the best one for patients treated with palliative intent. We found that the optimal treatment schedule depends, among others, on the strength of competition among various subclones in a tumor. The computational platform developed allows optimizing chemotherapy protocols, within admissible limits of toxicity, for palliative treatment of metastatic NSCLC. The simplicity of the method allows its application to chemotherapy optimization in different cancers.

## Author summary

Lung cancer is usually diagnosed at an advanced stage because of non-specific symptoms. The most common subtype of lung cancer is non-small cell lung cancer, which constitutes 80% of lung cancer cases. Here, we developed the methodology for finding the optimal treatment schedule for patients treated with palliative intent. The goal is not to cure the patients who are at an advanced stage but to prolong their survival by the administration of platinum-based chemotherapy. The method is based on the mathematical model describing the growth of tumors and its response to chemotherapy which is calibrated using real clinical data.

## Introduction

Resistance to treatment is a major challenge in oncology [1,2]. Even though the majority of patients initially respond to primary treatment, cancer relapse is frequently observed, sometimes after a short-time-interval [3]. One cause of treatment resistance is tumor heterogeneity and the mode of tumor evolution [4,5]. The treatment causes the death of cells that are sensitive and results in the selective advantage for resistant cells, which contribute to the residual disease and affect final outcome. As a result, when the tumor reoccurs, the patient is already resistant to drugs with similar model of action, i.e., multi-drug resistance is present.

Non-small cell lung cancer (NSCLC) is one of the most molecularly heterogeneous subtypes of cancer [6–8]. The heterogeneity exists on at least three levels: inter-patient, intra-patient, and intra-tumor. Inter-patient heterogeneity in NSCLC is due in part to the presence of cell subtypes: squamous, adenocarcinoma, and large-cell. Intra-patient heterogeneity is manifested by multiple primaries and dissemination of a primary tumors to distant organs [9]., intra-tumor heterogeneity has been proven through single-cell sequencing of lung cancer [10]. Tumor heterogeneity creates a challenge for treatment planning as well as prediction of response to treatment. Specifically, it is not known whether a patient will have a long-term response to administered treatment or a short-term one.

Treatment of lung cancer patients is usually a combination of chemotherapy, radiotherapy, and immunotherapy, as well as a targeted treatment such as for EGFR inhibitors. Currently, the standard of care for locally advanced NSCLC patients includes a combination of radio- and chemotherapy called chemoradiation. The metastatic NSCLC patients, until recently, were treated mostly with platinum doublet[11]. Here, we focus on the platinum doublet palliative treatment of non-small cell lung carcinoma.

Mathematical models could contribute to personalized cancer treatment through the application of optimization tools to determine the optimal dose and schedule of administration of an anticancer agent [12,13]. Three main strategies of drug scheduling have been developed empirically with theoretical suggestions from mathematical modeling. The first is the dose-dense therapy; involves frequent drug administration with a interval between two consecutive cycles shorter than three-weeks [14]. The second is metronomic therapy which involves frequent low-dose administration of a drug [15]. The last one is adaptive therapy in which the dose is adjusted based on response to treatment [16].

In the present paper, we apply a computational approach which includes a machine learning algorithm (MLA) for estimation of parameters of a mathematical model, this latter simulating the time-dependent counts of sensitive and resistant cancer cells in a NSCLC patient from diagnosis to death. The mathematical model we have developed is based on the models of lung cancer progression developed by Geng and collabolators [17] and Bajger and collaborators [18]. We extend the model by the inclusion of two types of cancer cell subpopulations sensitive and resistant to platinum-based chemotherapy.

Our main goal is to predict the response of patients treated with a palliative intent to chemotherapy involving a platinum doublet. In addition, we suggest a computational method to optimize the effectiveness of chemotherapy schedules used in therapy of advanced non-resectable NSCLC, based on a mathematical model that incorporates the effect of resistant cells selection.

## Results

### Application of mathematical modeling combined with MLA for prediction of response to anticancer treatment

We developed a method that combines mathematical mechanistic modeling with a machine learning algorithm to estimate response to anticancer treatment for each patient. The method is an extension of the approach suggested by Nicolò and colleagues [19]. The extension includes replacing calibration of the mechanistic model using mixed-effect learning with calibration mainly based on a multivariate Gaussian-mixture model.

The method uses patient clinical data as an input. As our goal is to predict initial and long-term response to palliative platinum doublet chemotherapy, we chose the following patient properties 1) overall survival (defining long-term response to treatment), 2) number of chemotherapy cycles administered to a patient (*CT_cycles_*), 3) time interval between two consecutive chemotherapy cycles (*T*), and 4) response to chemotherapy according to RECIST criteria (*CT_response_*) as an input to the developed computational framework.

**Fig 1:**
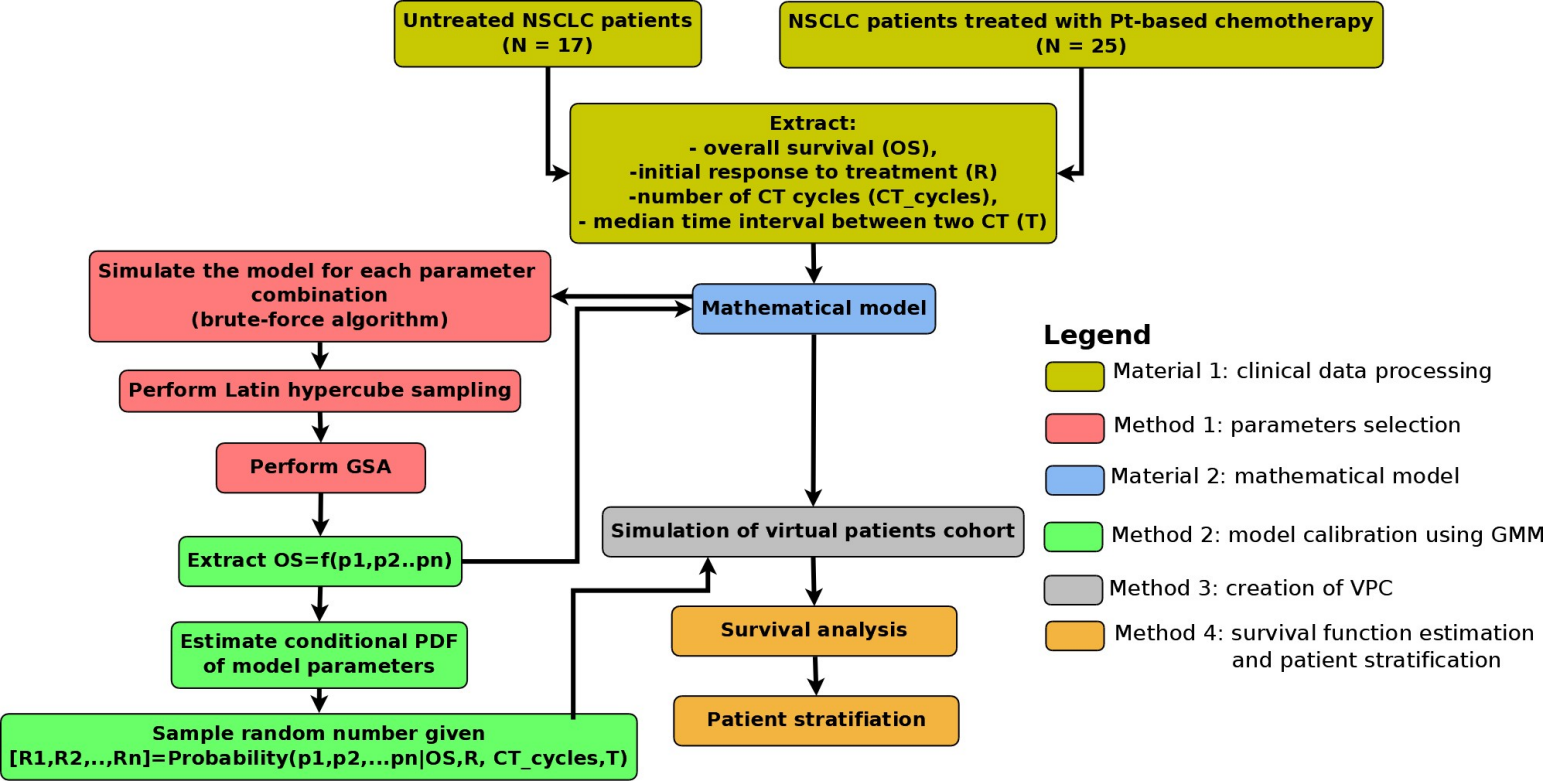
Computational framework. The diagram shows the flowchart of the computation. In the first step, the clinical data from NH and CT cohorts are merged. Next, the data are applied to estimate the probability density function (PDF) of *CT_cycles_* and T using Gaussian mixture model (GMM). In the next step, joint PDF, *P*(*DT,σ|OS*) is estimated using a combination of brute-force algorithm and GMM. The second and third steps provide the possibility to create virtual patients (VPs), who are then simulated from diagnosis until clinical death. In the last step, Kaplan-Meier analysis is performed and patients are stratified. The details of the computational framework are presented in the Materials and Methods section as well as in the Supplementary Text.

**Fig 2:**
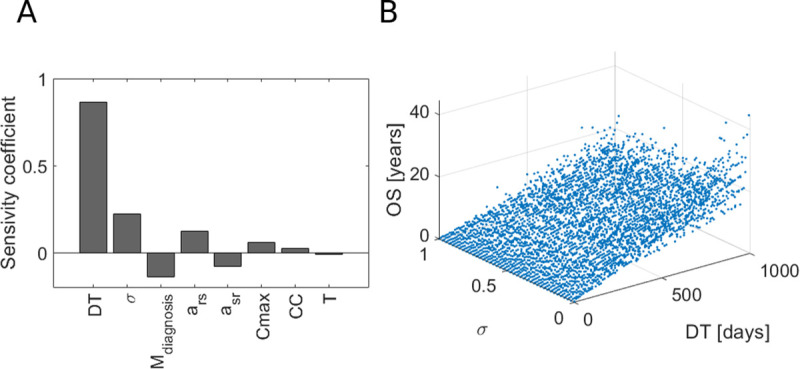
Selection of key model parameters affecting overall survival. A) Global sensitivity analysis (GSA) for eight model parameters with the overall survival as an output. B) Overall survival as a function of the two most sensitive parameters DT and σ.

The computational framework is depicted in Fig 1. The core of the proposed approach is creation of virtual patients (VPs), which includes the following steps. Firstly, global sensitivity analysis (GSA) is performed to extract the list of parameters that affect the output of our interest (here, the output is the overall survival). In our case, the most sensitive parameters are DT (doubling time) and σ (fraction of resistant cells at diagnosis) as shown in Fig 2A. Next, the mechanistic model is simulated for a wide range of sensitive parameters. In the third step, the clinical patients are bootstrapped and values of sensitive parameters for each bootstrapped patient are extracted. Next, a multivariate Gaussian mixture model (GMM) is trained using the expectation-maximization algorithm (E-M algorithm) implemented in MATLAB function *fitgmdist*. Finally, the parameters are sampled from the GMM using a random function in MATLAB environment. The sampled parameters define the virtual patient.

The method developed is very general as it can be applied to various patient cohorts. For example, it can be applied to predict not only response to chemotherapy, which is the focus of this paper, but also to radiotherapy or targeted treatment. Also, the applied machine learning algorithm and the mathematical model can be adjusted to a range of clinical questions and cancer types. Finally, the method can be applied to predict not only the long-term responses to anticancer treatment but also short-term responses such as disease-free survival (DFS) or progression-free survival (PFS).

### Tumor growth dynamics is a key variable affecting long-term response in NSCLC

As the first step in our computational platform, we performed global sensitivity analysis (GSA) to check, which parameter affects the most the long-term response in NSCLC patients. What is more important, here, the goal of GSA is selection of parameters which vary the most among the patients. This selection is a key method in the computational framework as it affects the downstream analysis and in particular determines which parameters define a virtual patient.

As shown in Fig 2A, the two most sensitive parameters are DT and σ. Thus, tumor growth dynamics is a key variable affecting overall survival. It is a counterintuitive result as we would expect that *M_diagnosis_* which directly describe tumor stage is a key variable. It means, that inherent growth dynamics, i.e. growth rate of sensitive and resistant cells, as well as tumor composition could possibly decide if the patient will have a short or long overall survival as depicted in Fig 2B.

### Calibration of the model to NSCLC patients treated with palliative intent

**Fig 3:**
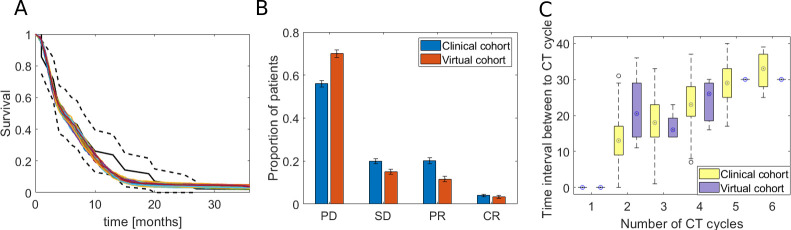
Calibration of the model to clinical data from NSCLC patients. A) Kaplan-Meier survival plot shows agreement between the virtual and clinical cohort. The black line shows the survival estimates for clinical data (solid line is an estimate, dotted lines define the 95% confidence interval). All other solid lines show Kaplan-Meier estimates for the virtual cohort (in total 100 cohorts with 1,000 patients each are shown). B) The plot shows agreement of the virtual cohort with a clinical one in terms of initial response. On the x-axis there is the initial response of patients by treatment effect: PD (progressive disease), SD (stable disease), PR (partial response) and CR (complete response) and on the y-axis there is proportion of patients in a cohort belonging to one of the four initial response class. C) The boxplot shows the relationship between the number of chemotherapy cycles (x-axis), and the time between the two consecutive chemotherapy cycles (y-axis).

Our method was applied to predict response of patients with unresectable non-small cell lung cancer to platinum doublet. Fig 3 shows the result of model calibration to the clinical cohort. We checked the agreement of the calibration on three layers of evidence 1) long-term response, 2) initial response, and 3) the relationship between the number of chemotherapy cycles and the time interval between cycles.

In Fig 3A, we show how the virtual cohort fits the clinical one with respect to a long-term response (overall survival, OS). We first fitted the non-parametric Kaplan-Meier model to the overall survival data in both clinical and virtual patients’ cohorts. Next, we performed two statistical tests (log-rank and Kolmogorov-Smirnov test) to check the agreement of the virtual OS with clinical ones. The log-rank test of the difference between the Kaplan-Meier (K-M) estimates of the OS values from the clinical and virtual cohort does not reject the hypothesis that the two K-M curves are identical (*p*>0.01). On the other hand, Kolmogorov-Smirnov statistical test does not reject the null hypothesis that the distribution of OS in virtual cohort is the identical as the one from the clinical cohort at a 5% significance level (*p* = 0.2639).

Initial response to chemotherapy was estimated using the RECIST criteria, which divide the patients into the ones with progressive disease (PD), stable disease (SD), partial response (PR), and complete response (CR). We divided the patients in our virtual cohort into these four groups according to the percentage of tumor reduction as a result of treatment (R). The criteria of patient stratification into PD, SD, PR, and CR groups in the virtual cohort are listed in Table 1. In brief, we assume that the initial response to chemotherapy is related to shrinkage of the tumor as a result of platinum doublet chemotherapy.

**Table 1:** Response criteria for virtual patients (VPs). R is the ratio of tumor burden before and after the treatment ($R=\frac{X_{s\_after}+X_{r\_after}}{X_{s\_before}+X_{r\_before}}$).

Response	criteria
**PD**	R ≥1.3
**SD**	0.9 ≤ R <1.3
**PR**	0.5≤R < 0.9
**CR**	R < 0.5

We show the agreement of the virtual and clinical cohort in the terms of the initial response to treatment in Fig 3B. We drew the bar plot with the fraction of patients who have the given initial response. To make the result reproducible, we simulated 100 virtual cohorts with 1,000 patients each as well as we generated 100 clinical cohorts with 1,000 patients using a bootstrapping method as explained in the Supplementary Text. As we can see, the initial response profile in the virtual cohort resembles the one in the clinical cohort. In addition, we performed the χ^2^ statistical test to check if the proportion of patients in each response group is similar in the virtual and clinical cohort. The test does not reject the null hypothesis at the 5% significance level (p = 0.99).

In the last layer of evidence that virtual cohort fit the clinical one, we checked if distribution of the time interval between two consecutive chemotherapy cycles as a function of chemotherapy cycles is indistinguishable. In Fig 3C we plotted the boxplots of T as a function of *CT_cycles_*. As we can see, Gaussian mixture model faithfully reproduces T as a function of *CT_cycles_*. The Supplementary Figure B in S1 Text shows the probability distribution function of PDF(DT, σ) and Supplementary Figure C shows the probability distribution function of PDF(*CT_cycles_,T*).

Next, to show that a minor subset of (DT, σ) pairs correspond to unique output (OS) values, we plotted, for each OS value, the PDF(DT, σ) in Supplementary Figure D in S1 Text. As we can see, only a narrow range of (DT, σ) fits the corresponding OS value and all pairs are clinically relevant.

### Initial response to platinum doublet does not correlate with a long-term response

**Fig 4:**
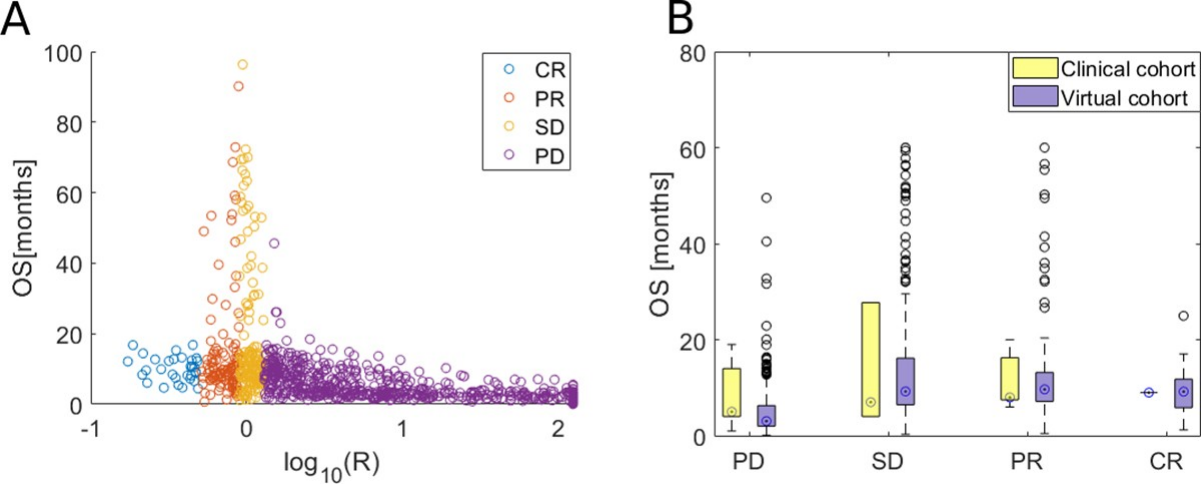
Long-term response to palliative platinum-doublet chemotherapy as a function of initial response. A) Initial response (R; log-transformed tumor reduction after treatment) versus long-term response (OS; overall survival in months) to platinum doublet chemotherapy in virtual cohort. Patients are divided into four groups CR (complete response), PR (partial response), SD (stable disease) and PD (progressive disease), based on the initial response to treatment (see Table 1). B) Overall survival as a function of initial response to platinum doublet chemotherapy in the clinical cohort.

To evaluate the relationship between initial response to chemotherapy and the long-term effect of treatment with the platinum doublet, we plotted the initial vs. long-term response. The initial response is plotted as a log-transformed tumor reduction by chemotherapy ($R=\log_{10}\left(\frac{X_{s\_after}+X_{r\_after}}{X_{s\_before}+X_{r\_before}}\right)$, whereas the long-term response is measured as OS in months. Fig 4A and Fig 4B show OS as a function of R correspondingly for the virtual and clinical cohorts. As in real patients, it is difficult to estimate the exact tumor reduction as a result of treatment intervention, for clinical cohort we divided patients into four categories according to RECIST criteria. As we can see in Fig 3A, the long-term response does not correlate with the initial one (Pearson correlation equals -0.45). It leads to the hypothesis that even if the patient has an excellent initial response, they might have short OS as a result of a competitive release of resistant cells [20]. Indeed, the mean fraction of resistant cells after treatment for patients with short OS and the good excellent initial response equals approximately 1.

In Fig 4B we depict the boxplots to show the relationship between initial (x-axis) and long-term (y-axis) response for virtual and clinical cohort. As we can see, the median OS (marked with a circle inside the box) is similar in each of initial response patient groups in both cohorts. It confirms our hypothesis that OS does not correlate with initial response (as hypothesized based on Fig 4A). Next, to further confirm the hypothesis, we perform the ANOVA statistical test, which confirms that mean OS between each initial response group is the same with a 95% significance level (p-value = 0.47).

### The optimal treatment schedule is dependent on competition coefficients

**Fig 5:**
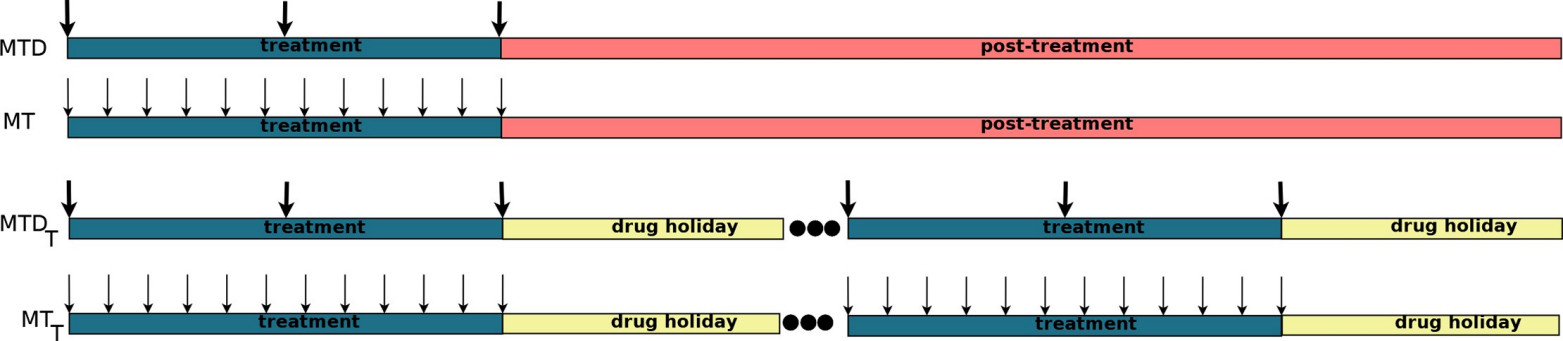
Treatment schedules considered in the palliative treatment of advanced non-resectable NSCLC patients.

Next, we performed computer simulations of virtual patients with four different treatment schedules to check which patients benefit from which type of treatment. This, in turn, allows the stratification of patients into the right treatment modality. As explained in the Materials and Methods section, we tested the following treatment schedules MTD (maximum-tolerated dose), MT (metronomic therapy), MTD_t_ (maximum-tolerated dose with a drug holiday), and MT_t_ (metronomic therapy with a drug holiday). Schematically, all four treatments are presented in Fig 5.

Each virtual patient is defined by four parameters DT, *σ*, CT_cycles,_ and T. The remaining parameters are constant. Each virtual patient is simulated using each treatment protocol described in Materials and Method. Each patient is treated eight times (MTD, MT, MTD_30_, MT_30_, MTD_60_, MT_60_, MTD_90_, MT_90_) where X_30_, X_60,_ and X_90_ is the treatment X with 30, 60 and 90 days of drug holiday, respectively. This type of analysis allows checking which patients benefit from which treatment schedule.

**Fig 6:**
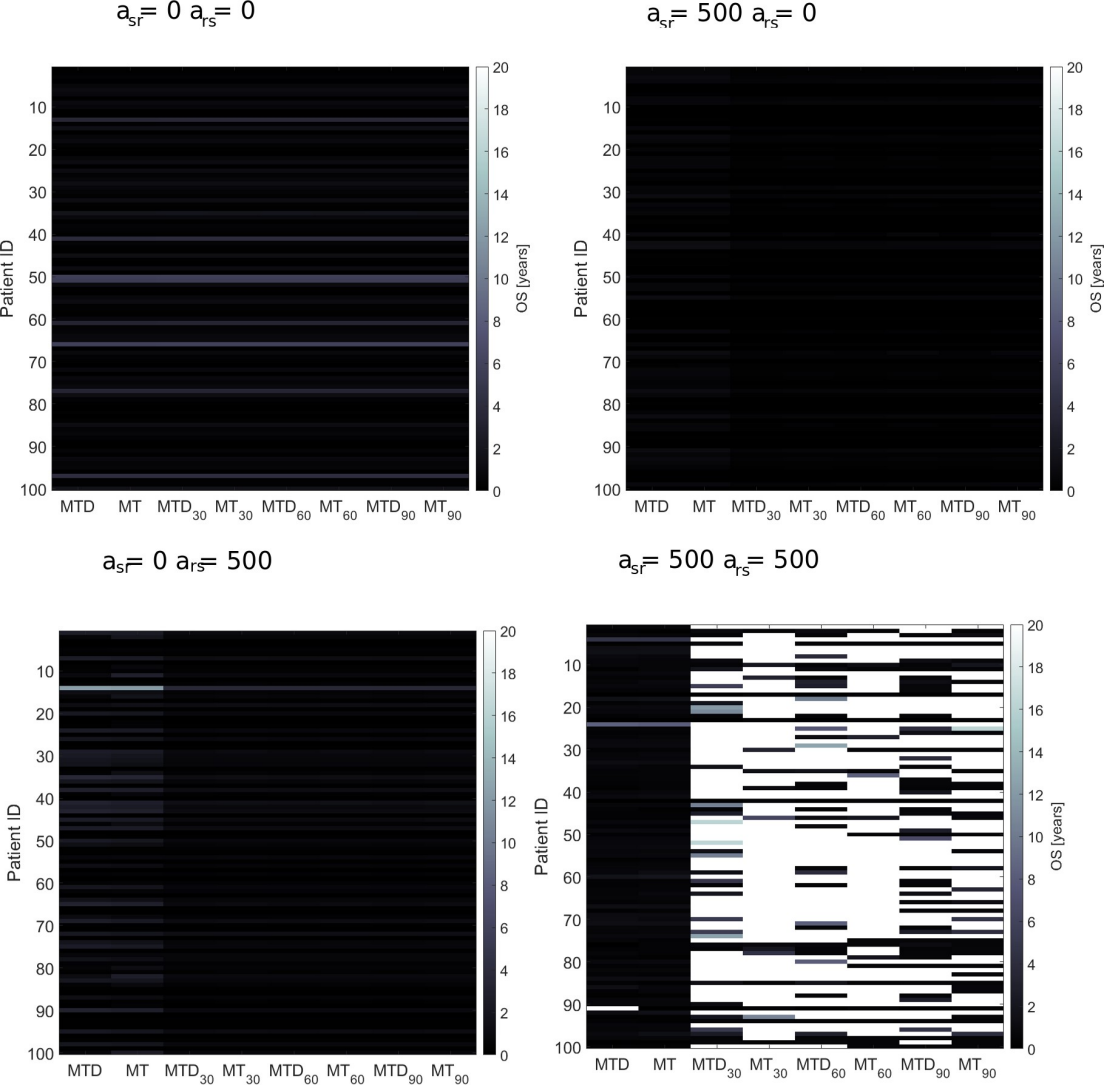
Comparison of all eight chemotherapy treatment schedules. For four combinations of a_sr_ and a_rs_, the heatmap shows the long-term response (represented by the colors) to palliative chemotherapy administered using eight different schedules. On x-axis we have different patient schedules and on y-axis patient ID. Optimal treatment schedule depends on dynamics of competition between various subclones in a tumor. For example, for high a_sr_ and a_rs_, the best outcome gives schedules with drug holidays, whereas for weak competition all schedule gives the same outcome.

The results of this analysis are presented in Fig 6. For four combinations of competition coefficients parameters (a_sr_ and a_rs_), we plotted the heatmap with patient ID on the y-axis, and treatment schedule on the x-axis. The color represents the overall survival of the virtual patient. Interestingly, for a_sr_ = a_rs_ = 0, all schedules result in a similar outcome. It means that in this case, doubling time and fraction of resistant cells at the diagnosis (described in the model with parameter DT and σ) divide the patients into those with short or long survival. No improvement is observed when drug holidays are incorporated because the resistant cells dominate in majority of patients with advanced non-resectable NSCLC as we showed by the model calibration.

However, in case *asr* = *ars* = 500, corresponding to a high competition between resistant and sensitive cells for space and resources, the best outcome occurs under schedules with drug holidays. Here, during the treatment phase, the number of sensitive cells decreases and the number of resistant cells increases, while during drug holidays it is reversed and the number of sensitive cells increases and the number of resistant ones (as a result of competition) decreases. When the competition is weak, the dynamics of sensitive and resistant cells resemble the case of no drug holidays. When the competition is moderate, we observed sustained oscillations of the sensitive and resistant cells if the drug holiday is set to optimal value. In the last scenario, when competition is high, we can observe slow shrinkage of resistant cells and outburst of sensitive cells. Here, the overall survival depends highly not only on DT and σ but also length of drug holiday and strength of competition between sensitive and resistant cells.

## Discussion

Drug resistance is one of the major causes of lung cancer death, and thus new chemotherapy treatment protocols to overcome treatment resistance are urgently needed. There exist several drug scheduling schemes, such as metronomic chemotherapy protocol, based on suggestions from mathematical modeling studies to tackle this clinical problem [15]. For example, metronomic therapy, which involves a low-dose frequent-time chemotherapy protocol, was tested in many clinical trials including breast[21], prostate [22], and lung cancer[23]. In most cases, mathematical models focus on the application of chemotherapy to lung cancer patients with a curative intent. However, a lot of clinical research in oncology focuses on patients with a poor prognosis who are treated in a palliative setting.

Accordingly, here we tackle the question of how to administer the platinum-doublet in a palliative life-extending setting, using a computational tool composed of MLA and of a mathematical model, taking into account toxicity limits. The goal is to suggest a novel protocol for the administration of platinum-doublet chemotherapy, which could be further tested in a clinical setting.

Using the computational approach, we first fitted the mathematical model of lung cancer by taking into account tumor heterogeneity and interaction between two major subclones, sensitive and resistant. Next, we created a virtual patient cohort (VPC) allowing stratification of patients based on four parameters doubling time, fraction of resistant cells at diagnosis, number of administered chemotherapy cycles, and time interval between two consecutive chemotherapy cycles. The patients in the group with good short- and long-term response to treatment are characterized by small fraction of resistant cells and long doubling time.

Next, we applied our method to find the best chemotherapy schedule for each individual patient. Interestingly, competition between sensitive and resistant cells play a key role in the patient stratification into the best chemotherapy schedules. For instance, patients with weak competition between subclones in the tumor benefit from all tested schedules equally. In contrast, patient with strong competition benefit significantly more from proposed by us schedules with drug holidays.

The herein proposed computational tool is suitable for a systematic testing of various protocols of chemotherapy administration in solid cancers. The platform allows first to integrate the clinical data, such as drug dose and survival information, with the mathematical model through MLA. Next, the tool can be applied to optimize the chemotherapy protocols by, for example, introduction of drug holidays into the treatment. Our approach based on MLA and mathematical modeling could be further extended by incorporation of additional effects such as interaction of tumor cells with tumor microenvironment which contains among other fibroblasts and immune cells.

As evident from Results, the modified schedules we propose lead, in simulations, to an increase of the overall survival time. This is due a strategy in which additional treatment is introduced, not “as needed”, i.e., when symptomatic tumor recurs, but periodically, in anticipation of the recurrence. Accordingly, the total dose is a multiplicity of the median total dose estimated from clinical practice (see Methods). It is assumed that it is acceptable if treatment repetitions are separated by a rather long hiatus, i.e., the “drug holidays”. How this strategy may work in practice, depends on many factors, which cannot be easily included in a model. They include, the unknown toxicity effects of periodic exposure, particularly the intertwined effects on bone marrow and immune system. The model predictions can be used as guidance for clinical trial design. The strategy we propose is not limited to classical chemotherapy. New therapies based on targeted agents are currently being introduced [24] and they offer, in principle, a lower toxicity. The cost-effective *in silico* approach to scheduling will be of use for such therapies too.

From optimal control theory point of view the proposed treatment protocol may be viewed as an approximate strategy of a singular solution to control optimization problem (see e.g. [25, 12]).

## Materials and methods

### Ethic statement

The study was approved by the Local Bioethical Commitee at Maria Sklodowska-Curie National Research Institute of Oncology, Gliwice Branch, Poland in accordance with national regulations. The approval was granted by the named board according to national regulations. A formal written consent was obtained from all participants of the study. The clinical data were anonymized before the computational analysis.

### Patient data

Patients included in the present study were diagnosed with advanced non-operable non-small cell lung cancer (NSCLC) between 2004–2014. All patients were treated at Maria Sklodowska-Curie National Research Institute of Oncology, Gliwice Branch, and followed on average for 3 years.

Out of 42 patients diagnosed with advanced non-resectable NSCLC who were treated with platinum-based chemotherapy or had symptoms treated, we extracted 42 cases with complete follow up. This included 17 individuals who received symptoms treatment only (natural history, NH, cohort) and 25 patients who underwent platinum-doublet chemotherapy (chemotherapy, CT, cohort). The data are presented in Table A and Table B in supplementary information, whereas the comparison of the two cohorts is presented in Table 1.

Table 2 compares the two groups. Patients in the NH group were older, with an average age at the diagnosis 67 versus 63 in the CT group. Zubrod performance score (WHO scale) was higher for the NH group as 30% of patients (versus 8%) had a Zubrod score equal to two [26]. It indicates that patients in the NH group had poor general performance, and thus chemotherapy could have been too toxic for those patients. Accordingly, the NH patients did not receive intensive treatment.

**Table 2:** Summary of patient cohort. Comparison of natural history (NH) cohort with chemotherapy (CT) cohort. ^1^Performance score–performance score using Zubrod scale, ^2^OS–overall survival, ^3^MFS–metastatic-free survival, ^4^response to CT–response to chemotherapy according to RECIST criteria.

		NH cohort	CT cohort
		N = 17	N = 25
Age		67(55–78)	63 (47–76)
Sex	Female	5 (30%)	5 (20%)
	Male	12 (70%)	20 (80%)
Performance score^1^	0	5 (30%)	1 (4%)
	1	7 (40%)	22 (88%)
	2	5 (30%)	2 (8%)
Stage	IIB	1 (6%)	0 (0%)
	IIIA	1 (6%)	12 (48%)
	IIIB	8 (47%)	8 (32%)
	IV	7 (40%)	5 (20%)
T	1	0 (0%)	1 (4%)
	2	2 (12%)	7 (28%)
	3	4 (24%)	7 (28%)
	4	11 (64%)	8 (32%)
	x	0 (0%)	2 (8%)
N	0	3 (18%)	1 (4%)
	1	1 (6%)	2 (8%)
	2	8 (47%)	12 (48%)
	3	4 (23%)	9 (36%)
	x	1 (6%)	1 (4%)
M	0	10 (60%)	20 (80%)
	1	7 (40%)	5 (20%)
Subtype	squamous	14 (82%)	16 (64%)
	adenocarcinoma	2 (12%)	5 (20%)
	other	1 (6%)	4 (16%)
OS^2^		7 (1–27)	12 (1–81)
MFS^3^		4 (0–27)	7 (0–42)
Chemotherapeutic drugs	cisplatin+ vinorelbine		22 (88%)
	cisplatin+gemicitabine		3 (12%)
Response to CT^4^	complete response		1(4%)
	partial response		5 (20%)
	stable disease		5 (20%)
	progressive disease		8(32%)
	undetermined		6 (24%)

Tumor stage was estimated according to the 7^th^ edition of AJCC[27]. Patients in the NH cohort had average stage IIIA and patients in CT group had average stage IIIB. In addition, most patients in NH had an advanced primary tumor (T3-T4). According to the TNM classification, most patients in both groups have advanced nodal stage (N2-N4). Distant metastases were detected in 40% of the NH cohort and 20% of the CT cohort. Patients in the NH group were more advanced, and thus, their prognosis was inferior compared to the CT group.

Patients in the CT cohort were treated with a platinum-based doublet. 84% of patients were treated with cisplatin combined with navelbine, and a small fraction of patients was treated with cisplatin combined with gemcitabine. The median of three cycles of chemotherapy every three weeks was administered. The patient's response to treatment was poor as most patients (52%) were classified as stable or progressive disease.

### Mathematical model of resistance to platinum doublet in non-small cell lung cancer

We adapted and further developed the model of dynamics of platinum-sensitive and platinum-resistant cells assuming that tumor is composed of different types of cells interacting with each other in a non-linear fashion [28,29]. Cancer cells compete for space and other resources, and their evolution results in some types of cells surviving and others dying out due to chemotherapy and selection pressure. The model describing dynamics of platinum-sensitive and resistant-cells and competition between them uses a version of the competition model, similar to models of Gatenby and collabolators[16,30,31]. We assume that both types of cells are growing according to logistic dynamics and interact with each other via clonal interference (see Gerrish and Lenski 1998) [32].

To model tumor growth in the presence of platinum-based chemotherapy, we assume that it affects only platinum-sensitive cells. The effect of chemotherapy is modeled according to the Norton-Simon hypothesis (N-S), which states that solid tumor cells are killed proportionally to the growth rate of the unperturbed tumor [33,34]. We incorporate pharmacokinetics of cisplatin into the model, with cisplatin assumed to be administered via intravenous bolus injection [35]. For simplicity, we assume no delay between cisplatin injection and the time cisplatin concentration reaches a maximum in cancer cells. Mathematically, pharmacokinetics dynamics corresponds to a one-compartment model. All model parameters for advanced non-resectable NSCLC are listed in Table 3 and key model assumptions are listed in Supplementary Text.

**Table 3:** List of parameters of the mathematical model for NSCLC patients.

symbol	value	description	reference
DT_s_	5–1000 days	Doubling time of sensitive cells	Fitted to clinical data
λ_s_	ln(2)/DT_s_[1/day]	Growth rate of sensitive cells	Fitted to clinical data
λ_r_	0.5 ·λ_s_[1/day]	Growth rate of res. cells	We assume that resistant cells grow twice smaller than sensitive ones
K	30 cm diameter	Carrying capacity	[17]
a_sr_	0	Competition coefficient	For model calibration we set the parameter to zero (for simplification of the model calibration). In other analyzes we simulated the model for various values of a_sr._
a_rs_	0	Competition coefficient	For model calibration we set the parameter to zero (for simplification of the model calibration). In other analyzes we simulated the model for various values of a_rs._
M_diagnosis_	4 cm diameter	Tumor burden at the diagnosis	[17]
σ	0–1	Fraction of resistant cells at diagnosis	Varied
M_death_	15 cm diameter	Lethal tumor burden	[36]
T	21 [days]	Time interval between two consecutive cycles of chemotherapy	Fitted to clinical data
k	0.211	Clearance rate of cisplatin	Half-life of cisplatin is 80 hours
C_max_	20 [A.U.]	Dose of chemotherapy	Fitted to clinical data
CT_cycles_	0–6	Number of chemotherapy cycles	Fitted to clinical data

The model is defined with the following system of coupled ordinary differential equations:

$$\dot{X_{s}}=\lambda_{s}\cdot X_{s}\left(1-\frac{X_{s}+a_{rs}{\cdot X}_{r}}{K}\right)(1-C(t))$$

$$\dot{X_{r}}=\lambda_{r}\cdot X_{r}\left(1-\frac{X_{r}+a_{sr}{\cdot X}_{s}}{K}\right)$$

Where X_s_ and X_r_ are amount of sensitive and resistant cells, respectively. Drug concentration, however, is modelled with the following algebraic equation:

$$C(t)=C_{0}\cdot exp(-k\cdot t)$$

where C_0_ is initial drug concentration which is equal C_max_ at the time of drug administration.

### Mathematical model calibration to patient data

The workflow of model calibration and model application is presented in Fig 1. It is composed of two Materials (clinical data and mathematical model) and four Methods. A detailed description of the framework is in Supplementary Text and the framework is presented schematically in Fig 1.

The core of the model calibration is the application of multivariate Gaussian Mixture model describing the conditional probability *P*(*p*_1_,*p*_2_,…,*p_n_|OS*) *where p*_1_,*p*_2_,…,*p_n_* are model parameters and *OS* is patient overall survival. First, we performed parameter selection using global sensitivity analysis (GSA) by choosing only those which are sensitive to OS. Next, we applied the Brute-Force algorithm to estimate *OS* = *f*(*p*_1_,*p*_2_,…,*p_n_*) through model simulations. In the third step, the conditional probability density function is estimated by fitting to the Gaussian Mixture Model (GMM) using the Expectation-Maximization algorithm. From the probability density function, the varied parameters are sampled and the virtual patient is defined with those varied parameters.

### Simulation of virtual patient cohorts

The core of our framework is the creation of virtual patients [37,38]. The model is a deterministic system of two coupled ordinary differential equations, but simulations are stochastic. Before the start of simulations, the parameters marked in Table 2 as varied, are sampled from a fitted multivariate Gaussian Mixture probability distribution using the standard random number generator in MATLAB.

The model is then simulated for each virtual patient from the time of diagnosis until death. The model is simulated using Runge-Kutta numerical method for solving the differential equations (ode45 function in MATLAB environment). From model simulations, overall survival (OS) and initial response to treatment (R) are extracted. The details of model simulations are described in Supplementary Text.

### Treatment schedules

We performed model simulations using the following protocols: 1) maximum-tolerated dose schedule, 2) maximum-tolerated dose schedule with drug holiday, 3) metronomic therapy schedule, and 4) metronomic therapy with drug holiday. Graphically, the schedules are presented in Fig 5.

The maximum-tolerated dose (MTD) therapy is simulated as follows [39]. The virtual patient after diagnosis and without time delay is treated with three cycles of cisplatinum with dose 80mg/m^2^ and 21 days’ time interval between each two consecutive cycles. Thus, the total dose of chemotherapy is 240 mg/m^2^ which is below the threshold of cisplatin toxicity (which is 600 mg/m^2^) [40]. None of the virtual patients treated with MTD schedule receives secondary treatment. We simulate metronomic therapy (MT), which is also called the low-dose frequent-time therapy, similarly to MTD ^15^. The difference is that for MT, the frequency is *T* = 5 days and dosage of cisplatinum is 20 mg/m^2^.

Next, we suggest the MTD and MT with drug holidays. The protocol is aimed at reducing cisplatin toxicity by allowing the virtual patient to rest from treatment which helps to restore proper values of blood cell count. In the MTD protocol, the sequence of chemotherapy cycles is administered with dose 80mg/m^2^ and time interval between two consecutive cycles of 21 days until total dose reaches 600 mg/m^2^. Drug holiday (when no treatment is administered) follows the sequence of chemotherapy cycles. After the first drug holiday, next sequence of chemotherapy cycles is administered, followed by another drug holiday. The treatment is continued over patient’s lifetime. In case of MT with drug holiday the process is similar. Similarly, as in the scenario without drug holidays, for the MT, the frequency is *T* = 5 days and dosage of cisplatinum is 20 mg/m^2^.

## Supporting information

S1 Text Description of the computational platform. (PDF)
