# Supplementary material for: Mathematical model predicts response to chemotherapy in advanced non-resectable non-small cell lung cancer patients treated with platinum-based doublet
Source: PLoS Comput Biol. 2020 Oct 5;16(10):e1008234. doi: 10.1371/journal.pcbi.1008234 (PMC7561182; doi:10.1371/journal.pcbi.1008234)
Supplement: S1 Text — (PDF) [file pcbi.1008234.s001.pdf]

Supplementary material

Mathematical model predicts response to chemotherapy in advanced non-resectable non-small cell lung cancer patients treated with platinum-based doublet

Emilia Kozłowska, Rafał Suwiński, Monika Giglok, Andrzej Świerniak, Marek Kimmel

Date: September 28, 2020

Contents

Text A. Computational platform 2
Material 1. Clinical data 2
Material 2: Mathematical model 3
Method l: Parameters selection 4
Method 2: Model calibration using Gaussian Mixture Model 6
Method 3: Creation of a virtual patient cohort 6
Method 4: Survival analysis and patient stratification 7

Text B. Mathematical model simulation 8

Table B. NH cohort 10

Table C. CT cohort 11

Figure A. Global sensitivity analysis 12

Figure B. Probability density function of (DT, σ) 13

Figure C. Probability density function of (CT_{cycle}, T) 14

Figure D. Probability density function of (DT, σ) for each clinical patient 15

Text A. Computational platform

The developed computational platform is composed of a mathematical model and its calibration, virtual patients (VP) cohort, and patient stratification and treatment optimization. The input data to the developed platform are clinical data. The data are first pre-processed to extract the necessary information for model calibration such as overall survival, treatment protocol (dose and frequency of chemotherapy), and tumor stage.

The core of the platform is the generation of virtual patients (VPs) as explained in the main text. The creation of VPs is based on sampling values of selected parameters from probability density function estimated using clinical data. Next, the deterministic model in the form of ordinary differential equations is simulated using a numerical method (here, we use the Runge-Kutta 4-5 method). Thus, the patients are defined with a set of parameter values that results in a different clinical outcome for each patient.

The computational framework is modular as each element in the platform can be adjusted or changed based on clinical questions and cancer type of interest. The platform is implemented in the MATLAB environment as it is one of the most popular scientific computation environment applied in mathematical modeling because of the efficient implementation of algorithms for solving differential equations. The MATLAB implementation of the computational framework is available on GitHub under the following address `https://github.com/EmiliaKo/PalliativeTreatmentNSCLC`.

Material 1. Clinical data

The clinical cohort includes 47 patients with non-small cell lung carcinoma (NSCLC) who were diagnosed at the 2nd Radiotherapy and Chemotherapy Clinic, M. Sklodowska-Curie National Research Institute of Oncology, Gliwice Branch, in Poland between 2004-2014. Patients were divided into two clinical arms: natural history (NH) and chemotherapy (CT). NH patients receive only symptomatic treatment, whereas CT patients receive chemotherapy in the form of the platinum doublet. The clinical data of NH patients are presented in Supplementary Table A, whereas clinical data of CT patients are shown in Supplementary Table B.

Dataset 1: Natural history cohort (NH cohort)

The cohort includes 17 patients with NSCLC (14 patients with squamous-cell cancer, 2 with adenocarcinoma, and 1 with another subtype), which are inoperable due to: i) patient age, ii) tumor location, or iii) general patient performance. These patients have a very poor prognosis as their cancer is at an advanced stage. Because of it, and patients will, all the patients in the cohort do not receive treatment in the form of chemotherapy, targeted therapy, or radiotherapy. Thus, this group of patients represents the natural history of advanced NSCLC.

Dominant patient sex is male (70% of patients). It represents the fact that the incidence of lung cancer is higher in males as they are more often smoking cigarettes. The average patient age is 67 years at diagnosis. At this age, risks of surgery are higher than average, particularly that smoking compromises the cardiovascular system. The performance score for each patient was determined using the Zubrod scale [7]. Interestingly, the higher Zubrod score in the NH cohort is two. It means that at the time of diagnosis, patients usually can take care of themselves.

Patients are diagnosed at an advanced stage with 87% of patients having a tumor of stage IIIB or IV, according to AJCC staging. As indicated by TNM classification, patients have a primary tumor size above 7 [cm] at the highest dimension. Around 70% of patients have metastasis to lymph nodes at diagnosis. Also, 40% of patients have distant metastases at the time of diagnosis. The most common places of distant metastases in the patients from the NH cohort are the brain, bones, and liver.

Dataset 2: Chemotherapy cohort (CT cohort)

The cohort includes 25 patients with NSCLC (16 patients with squamous cell carcinoma, 5 with adenocarcinoma, and 4 with other subtypes), which are unresectable similarly the patients in the NH group. These patients also have a very poor prognosis, but according to the patient's condition and consent, they were treated with the platinum doublet.

Dominant patient sex is male (80% of patients), which is very similar in the NH cohort. Patients are also elderly as their average age at the diagnosis is 63 years. Interestingly, the patient's performance score is slightly higher than in the NH cohort as most of the patients have the Zubrod score equal to one.

Half of the patients in the CT cohort are diagnosed at a highly advanced stage (stage IIIB or IV) and a half with advanced stage (IIIA). According to TNM classification, 50% of patients in the CT cohort have a primary tumor above 7[cm] (T classification). Most of the patients (95%) have lymph nodes metastases (N classification), and 20% have distant metastases (M classification).

Patients in the CT cohort received the treatment with cisplatin, usually combined with navelbine. A small fraction of patients is treated with cisplatin combined with gemcitabine or vinorelbine. Patient response to treatment is poor as only 4% of patients have complete response compared to 32% of patients with progressive disease. The reason for the poor response is the fact that patients in the CT cohort are treated with a palliative intent. The patients received a median of three cycles of chemotherapy. Patients received at minimum one cycle of chemotherapy and a maximum of six cycles. The median time interval between two consecutive chemotherapy cycles is 17 days. Patients receive on average a cisplatin dose of 80 $\left[mg/m^2\right]$ per chemotherapy cycle.

Material 2: Mathematical model

The mathematical model developed describes the dynamics of platinum-sensitive and platinum-resistant cells with and without treatment intervention. In addition to modeling the growth of cancer cells, we also model pharmacokinetics of platinum-based chemotherapy. We described the model in the form of a system of coupled ordinary differential equations (ODEs).

Growth of treatment sensitive and resistant cells is modeled using a logistic growth model with growth rate equal to λ_s and λ_r for sensitive and resistant cells, respectively. The growth rates are decreasing logistically until the number of cells reaches carrying capacity K. This allows for more realistic system formulation as in reality tumor cells are not growing exponentially but rather their growth is restricted by the surrounding tissues.

In the model, sensitive cells are characterized by a full response to platinum-based chemotherapy which is included in the model according to the Norton-Simon hypothesis (N-S) which states that the effect of chemotherapy is proportional to the unperturbed tumor growth. Mathematically, the N-S hypothesis could be formulated as:

$$\dot{X} = f(X) \cdot (1 - C(t)) \tag{A}$$

where $f(X)$ is a function describing the tumor growth and $C(t)$ is a drug concentration at time t. Resistant cells, in contrast, fully do not respond to chemotherapy. Thus, they have $C(t) = 0$ during the simulations. To keep the model simple and the number of model parameters small, we decided disregard the partially-resistant cells. This, allows us to create a model with identifiable parameters.

What is important, we also included in the model of tumor growth, the competition of tumor cells for resources and space. It is included by reduction of the growth rate of sensitive cells by resistant ones (through parameter a_{rs}) and reduction of the growth rate of resistant cells by sensitive ones (through parameter a_{sr}).

An important part of the model is pharmacokinetics of platinum-based chemotherapy which is represented by a one-compartmental model describing the exponential decay of drug concentration. The

drug administration in all our simulations is pulsed, which reflects the reality that the chemotherapy is administered as an intravenous bolus injection.

Key assumptions of the model

The mathematical model is governed by the following key assumptions:

1. Two types of cells are included in the model platinum-sensitive and platinum-resistant.
2. Partially-resistant cells are not included in the model for the sake of simplicity.
3. Both types of cells, platinum-sensitive and platinum-resistant ones, are growing according to logistic growth law.
4. Two types of cells included in the model compete for resources and space in a non-linear fashion.
5. Only innate platinum resistance is included in the model as it is the dominant one in lung cancer.
6. At the time of diagnosis, a small fraction of cells in the tumor is resistant to platinum.
7. Pharmacokinetics of cisplatin is modeled as a one-compartmental model with exponential decay of cisplatin concentration.
8. Cisplatin kills only sensitive cells, and resistant ones are left intact.
9. Cisplatin toxicity constraints are not explicitly included in the model.
10. Clinical lung cancer diagnosis is made when the tumor burden reaches $M_{diagnosis}$ cells.
11. Patient death is observed when tumor burden reaches M_{death} cells.
12. M_{death} equals half of carrying capacity $M_{death} = 0.5K$.
13. $M_{diagnosis} < M_{death}$, thus diagnosis is before patient death.
14. After relapse, no second-line treatment is administered to the virtual patients.

Method l: Parameters selection

The method is composed of two steps, the model simulations for all parameter combinations of interest and global sensitivity analysis (GSA). Below, we describe both steps in detail. The goal of this Method is selection of the parameters, which affect the long-term response to platinum doublet chemotherapy the most. This, in turn, helps us to chose the most important model parameters which vary among patients in a clinical cohort and therefore should be varied among patients in a virtual cohort.

To explore the dependence of model parameters on the overall survival, we performed the model simulation for a wide range of parameter values. It is performed in three steps, choosing the parameters ranges, the performance of Latin hypercube sampling (LHS), and the model simulation of virtual patients from cancer diagnosis until death for each parameter combination.

Selecting the parameter ranges

The parameter ranges are chosen that they span all possible values for lung cancer or, in case it is impossible, for a solid tumor. The list of parameters together with their ranges are listed in Table A. Three out of eleven parameters are set to constant values to minimize the possibility that the given set of parameters is not clinically relevant. Thus, we assume that the cisplatin elimination rate, lethal tumor burden as well as carrying capacity are constant, leaving eight parameters which could vary among patients. In the case of three parameters, the ranges of their values are assumed because they are not measurable. Indeed, C_{max} and competition coefficient are difficult to measure in real patients as well as *in vitro* or *in vivo*.

Table A: Ranges of model parameters values

parameter	parameter range	constrains	reference
DT	$5 - 1000\ days$		[1]
a_{sr}	$0 - 500$	Assumed	
a_{rs}	$0 - 500$	Assumed	
K	$30\ cm$	$K = 2 \cdot M_{death}$	[4]
$M_{diagnosis}$	$1 - 7; cm$	taken from TNM classification	[5]
M_{death}	$15\ cm$	$M_{death} = 0.5 \cdot K$	[2]
σ	$0 - 1$	Tumor can be fully-resistant at diagnosis	
C_{max}	$0 - 40[a.u.]$	Assumed	
T	$7 - 40\ days$	Clinical data	
CT_{cycles}	$1 - 6$	Clinical data	
k	0.211	Half-life of cisplatin equals to 80 [hours]	[8]

Perform Latin hypercube sampling

In the second step, Latin hypercube sampling is performed to sample the parameter values evenly. In short, the LHS method rests on dividing the parameter ranges for a given parameter into N equal small ranges, where N is the number of parameter combinations we are interested to sample. Next, within each smaller range, one parameter set is sampled from a uniform distribution. In total, we performed a sampling of 10,000 parameter combinations. As a result, we obtain an evenly distributed parameter values which are independent of each other. In practice, the LHS was performed in the project using the lhsdesign function in MATLAB environment. LHS allows us to cover the values of parameter ranges uniformly.

Perform global sensitivity analysis

The global sensitivity analysis was performed as follows. Having simulated the model with a wide range of parameter values, we first plotted overall survival as a function of each parameter separately (see Figure A). As we can see visually, the parameter, which linearly affects overall survival is DT. It is expected that overall survival in the computer simulations is calculated as a time until the tumor reaches a certain (lethal) size.

Next, to quantify the dependence of each parameter on overall survival, we compute the Pearson correlation coefficient between each parameter and overall survival. As a result, we have a list of parameters that correlate with OS, i.e., a list of sensitive parameters. The values of the correlation coefficient for each mathematical model are depicted in Figure 2 of the main text. Based on Figure 2, we chose two parameters DT and σ as parameters that could control the overall survival in the developed mechanistic model.

Method 2: Model calibration using Gaussian Mixture Model

The goal of this Method is to estimate the values of parameters, which vary from patient to patient. To choose the values of each parameter of choice, we need the probability distribution functions of the parameters allowing us to sample the parameter values.

Estimation of OS as a function of the model parameters

In the first step, we consider the function: $OS = f(p_1, p_2, \ldots, p_n)$, where $p_1, p_2, \ldots, p_n$ are parameters we chose using Method 1. The function is estimated as follows. Firstly, function $OS = f(CT_{cycles}, T)$ is estimated using the clinical cohort. Here, the pair $f(CT_{cycles}, T)$ from clinical data is bootstrapped with replacement 10,000 times giving us a large clinical cohort. Bootstrapping is performed as the number of patients in the clinical cohort is relatively small. Next, the bootstrapped values serve as an input to the Expectation-Maximization algorithm applied to estimate the parameter values of the Gaussian Mixture Model (GMM). The model parameters, which could be directly extracted from clinical data are sampled from the fitted Mixture Gaussian Model.

In the next step, multivariate probability density function ($PDF(DT, \sigma)$) is estimated using the Gaussian Mixture model with the results from simulations as an input. Here, firstly, the model parameters are selected uniformly using the ngrid function in MATLAB environment and, for each parameter set, the model is simulated. In total, we performed simulations for 4,100 parameter sets to extract overall survival. It is important to mention that we vary only parameters of choice (see Method 1) and the rest of the parameters are extracted from literature (see Table 2 in the main text). The simulations allows estimating $OS = f(DT, \sigma)$.

Estimation of conditional probability distribution

Here, we estimate the conditional probability dnsity function ($PDF(DT, \sigma, C_{max}|OS, R)$) of DT and σ conditional on long-term (OS) and short-term (R) response to platinum doublet chemotherapy.

Firstly, the value of C_{max} is chosen and set as a parameter with a constant value during the whole simulation and equal for each virtual patient. Next, the clinical patients (OS) are bootstrapped with replacement. Next, for each bootstrapped patient, every parameter combination leading to the same OS as the one observed in the bootstrapped patient. As a result, we have a list of parameter combinations that fit the individual patient (see Supplementary Figure B). From the list, one combination is taken with an equal probability for each combination. As a result, we estimated OS as a function of DT and σ.

In the second step, we fitted DT and σ to the multivariate Gaussian Mixture model (GMM) using the expectation-maximization algorithm. This, allows us to estimate multivariate probability density function of (DT, σ). The estimated probability density function is shown in Supplementary Figure C.

Next, the fitness of the model to short-term response is evaluated as follows. From a clinical cohort, the proportion of patients with a given initial response (stratified into CR, PR, SD, and PD group) is extracted. The same procedure is performed also for the virtual patient cohort by patient stratification into CR, PR, SD, and PD by R as depicted in Table 1 of the main text. Next, the proportions between clinical and virtual cohort are compared with the χ^2 statistical test.

All steps described in this section are repeated until the χ^2 statistical test accepts the null hypothesis that the initial response to platinum doublet chemotherapy is the same in both clinical and virtual cohort with a significant level equal to 5%. In such a way, we estimated $C_{max} = 20[A.U.]$.

Method 3: Creation of a virtual patient cohort

The method for the creation of virtual patients is composed of two steps generation of (DT, σ) and (CT_{cycles}, T) as well as simulation of the mechanistic model.

Firstly, we make assumption that DT and σ is not dependent on CT_{cycles} and T. The rationale for this is the fact that the first two parameters affect long-term response and the last two the initial response to chemotherapy. Also, the first two parameters are directly extracted from clinical data whereas the last two are hard to extract from patients. The values of the first two parameters are sampled from the trimmed probability density function of GMM (see Method 2) using random function in MATLAB environment. The trimming is performed in such a way that the values generated by the GMM are within a certain range (see Method 1). The same procedure is performed for two last parameters. As a result, we have a virtual patient defined with four parameters DT, σ, CT_{cycles}, and T. In total 1,000 parameter combinations are sampled leading to 1,000 VPs.

Next, for each parameter combination, the mechanistic model described with a set of coupled differential equations 1-3 is simulated using the Runge-Kutta 4-5 numerical method (using ode45 function in MATLAB environment). The simulations are performed to extract the dynamics of sensitive and resistant cancer cells from the time of diagnosis until patient death. Details of the simulations are explained in Supplementary Text 2. As an output from simulations, we extract overall survival and initial response defined as a ration between tumor volume after and before platinum doublet chemotherapy.

Method 4: Survival analysis and patient stratification

To evaluate the fit of the virtual patient cohort to the NH cohort, we performed Kaplan-Meier (K-M) analysis. This analysis is routinely applied by clinicians to estimate survival probability as a function of time from diagnosis. Also, the K-M model is used for the comparison of different treatment arms in clinical trials.

The K-M analysis was performed using an ecdf function in MATLAB environment. Both overall survival from data, and model is done without censoring. The reason for this is that in model simulations, we do not consider patients who left the clinical study before the end of the follow-up period. Also, in the clinical cohort, we have only those patients with full history, i.e. the patient dies before the end of the follow-up period. The estimated survival function is presented in Figure 3 of the main text.

Next, we performed log-rank and two-sided Kolmogorov–Smirnoff statistical test. The first test is applied to compare two survival plots, whereas the second one examines the distribution of overall survival. The goal of these tests is to measure qualitatively if long-term responses between real and virtual patients are the same.

Text B. Mathematical model simulation

All mathematical model simulations are performed in MATLAB 2019 environment. As the model is non-linear, we performed numerical simulations by solving a system of ODEs. System of coupled ordinary differential equations is solved using Runge-Kutta method 4-5. Thus, the ode45 function in MATLAB environment was applied.

Model simulations without treatment (when $CT_{cycles} = 0$ and $T = 0$), the simulations are performed in one phase. Simply, the model is simulated from the time of clinical diagnosis until death. The tumor is diagnosed when the tumor diameter equals 4 cm. It corresponds to the primary tumor burden observed for very advanced stage NSCLC patients according to TNM classification. The model simulations are stopped, however, when the tumor burden reaches 15 cm in diameter. Thus, we assume that the primary tumor burden is a reason for patient death. The assumption is based on the fact that the tumor burden is a single clinical parameter, which causes death in the majority of patients [6].

Model simulations of patients treated with maximum-dose therapy (MTD) or metronomic therapy (MT) are performed in two phases treatment and post-treatment. The treatment phase is performed in such a way that the chemotherapy cycle is administered as an intravenous bolus injection (IV bolus). In the model, it is performed by increasing C by C_{max}. The pulses of chemotherapy are performed with a T time interval between two chemotherapy cycles and until CT_{cycles} pulses are administered. After the treatment phase, the model is simulated without any perturbation, i.e., C is not increased during whole post-treatment simulations. The simulations are stopped, similarly to the case when no treatment is administered, when the tumor burden reaches the lethal one.

The model simulations, in case drug holidays are incorporated, are performed sequentially. The sequence is composed of CT_{cycles} cycles of platinum doublet chemotherapy and time free of treatment lasting for $T_{drugholiday}$ days. The sequence is repeated until virtual patient death. This drug scheduling resembles so-called adaptive therapy [3]. The main difference is the type of drug holidays. Here, the drug holiday is fixed for each patient and is equal during the whole course of simulations. In adaptive therapy, the drug holiday is adapted depending on the response to the treatment.

From model simulations, two clinical outputs are extracted initial response to platinum doublet chemotherapy and overall survival. The initial response is computed as $\frac{X_s+X_r}{Y_s+Y_r}$, where $X(Y)$ corresponds to tumor burden at the end of treatment phase (before treatment). Overall survival of virtual patients is computed as a time interval between the start and end of the simulation,i.e., measured as a time elapsed from clinical diagnosis until death.

References

[1] T Arai, T Kuroishi, Y Saito, Y Kurita, T Naruke, and M Kaneko. Tumor doubling time and prognosis in lung cancer patients: evaluation from chest films and clinical follow-up study. Japanese Lung Cancer Screening Research Group. *Japanese journal of clinical oncology*, 24(4):199–204, aug 1994.

[2] Frank C. Detterbeck and Christopher J. Gibson. Turning gray: The natural history of lung cancer over time. *Journal of Thoracic Oncology*, 3(7):781–792, 2008.

[3] Robert A Gatenby, Ariosto S Silva, Robert J Gillies, and B Roy Frieden. Adaptive therapy. *Cancer Research*, 69(11):4894–4903, jun 2009.

[4] Changran Geng, Harald Paganetti, and Clemens Grassberger. Prediction of Treatment Response for Combined Chemo- and Radiation Therapy for Non-Small Cell Lung Cancer Patients Using a Bio-Mathematical Model. *Scientific Reports*, 7(1):13542, dec 2017.

[5] Peter Goldstraw, John Crowley, Kari Chansky, Dorothy J. Giroux, Patti A. Groome, Ramon Rami-Porta, Pieter E. Postmus, Valerie Rusch, and Leslie Sobin. The IASLC lung cancer staging project: Proposals for the revision of the TNM stage groupings in the forthcoming (seventh) edition of the TNM classification of malignant tumours. *Journal of Thoracic Oncology*, 2(8):706–714, aug 2007.

[6] Larry Nichols, Rachel Saunders, and Friedrich D Knollmann. Causes of death of patients with lung cancer. In *Archives of Pathology and Laboratory Medicine*, volume 136, pages 1552–1557, 2012.

[7] M. M. Oken, R. H. Creech, and T. E. Davis. Toxicology and response criteria of the Eastern Cooperative Oncology Group. *American Journal of Clinical Oncology: Cancer Clinical Trials*, 5(6):649–655, 1982.

[8] G. Panteix, A. Beaujard, F. Garbit, C. Chaduiron-Faye, M. Guillaumont, F. Gilly, P. Baltassat, and F. Bressolle. Population pharmacokinetics of cisplatin in patients with advanced ovarian cancer during intraperitoneal hyperthermia chemotherapy. *Anticancer Research*, 22(2 B):1329–1336, 2002.

Table B. NH cohort

Table B: Subtype- subtype of NSCLC (S - squamous, A - adenocarcinoma, O - other), ZS - Zubrod performance score, TNM - TNM classification, Loc - location (left/right lung), MFS - metastasis-free survival, MFSc - censoring for MFS, OS - overall survival, OSc - censoring for OS.

	Sex	Age	Subtype	ZS	Stage	TNM	Loc	MFS	MFSc	OS	OSc
1	M	55	S	1	IV	T4 N3 M1	R	1	1	1	1
2	M	68	S	1	IV	T3 N2 M1	R	0	1	2	1
3	M	61	S	0	IIIB	T4 N2 M0	R	25	0	25	1
4	M	71	S	0	IIIB	T4 N0 M0	R	1	0	1	1
5	M	66	S	0	IIB	T3 N0 M0	R	5	0	5	1
6	M	65	S	1	IV	T4 Nx M1	R	1	1	3	1
7	F	74	S	1	IIIB	T4 N2 M0	R	1	0	1	1
8	M	63	S	2	IV	T2 N2 M1	R	0	1	20	1
9	F	58	S	1	IIIB	T4 N2 M0	R	3	0	3	1
10	F	75	S	2	IIIB	T4 N2 M0	R	4	0	4	1
11	F	78	S	2	IIIB	T4 N2 M0	L	2	0	2	1
12	M	63	S	2	IV	T3 N3 M1	R	0	1	8	1
13	M	73	A	2	IV	T4 N2 M1	L	0	1	1	1
14	M	70	S	0	IIB	T3 N0 M0	L	27	0	27	1
15	M	71	O	0	IV	T2 N3 M1	R	0	1	1	1
16	M	71	S	1	IIIA	T4 N1 M0	L	1	1	9	1
17	F	64	A	1	IIIB	T4 N3 M0	L	2	0	2	1

Table C. CT cohort

Table C: Subtype- a subtype of NSCLC (S - squamous, A - adenocarcinoma, O - other), ZS - Zubrod performance score, TNM - TNM classification, Loc - location (left/right lung), Tint - the time interval between two CT cycles in days, CTcycles- the amount of CT cycles, Drugs - CT drugs combination (P+G -cisplatin+gemicitabine, P+V - cisplatin+vinorelbine), R- response to CT MFS - metastasis-free survival, MFSc - censoring for MFS, OS - overall survival, OSc - censoring for OS.

	Sex	Age	Subtype	ZS	Stage	TNM	Loc	Tint	CT_{cycles}	Drugs	R	MFS	MFS_{cens}	OS	OS_{cens}
1	M	63	S	1	IIIA	T3 N2 M0	R	30	6	P+V	PR	8	0	8	1
2	M	65	S	1	IIIA	T3 N2 M0	L	30	4	P+V	PD	14	0	14	1
3	M	68	S	1	IIIA	T2 N2 M0	R	23	3	P+V	NA	4	0	4	1
4	M	57	S	1	IIIA	T2 N2 M0	R	16	3	P+V	SD	4	0	4	1
5	F	54	A	1	IIIB	Tx N3 M0	R	17	3	P+V	PR	8	0	8	1
6	M	61	A	1	IIIA	T2 N2 M0	R	19	3	P+V	SD	5	1	10	1
7	M	62	S	1	IIIB	T4 N2 M0	L	20	3	P+V	PD	4	0	4	1
8	F	69	O	1	IV	T3 N3 M1	L	15	2	P+G	PD	0	1	4	1
9	F	65	S	1	IIIA	Tx N2 M0	L	36	2	p+V	PR	6	1	20	1
10	M	47	S	0	IIIB	T4 Nx M0	R	16	3	P+G	SD	4	0	4	1
11	M	60	S	1	IIIA	T2 N2 M0	L	30	5	P+V	PD	10	0	10	1
12	M	71	S	1	IIIA	T3 N2 M0	L	0	1	P+V	PD	2	1	13	1
13	M	64	S	1	IIIB	T2 N3 M0	R	11	2	P+V	PR	2	1	6	1
14	M	59	O	1	IIIA	T2 N2 M0	R	29	2	P+V	SD	42	1	81	1
15	M	50	A	2	IV	T4 N3 M1	L	16	4	P+G	PD	0	1	3	1
16	M	62	S	2	IIIA	T4 N1 M0	L	14	3	P+V	CR	8	1	9	1
17	M	76	S	1	IV	T1 N3 M1	R	14	3	P+V	PD	4	1	19	1
18	M	63	A	1	IIIB	T3 N3 M0	R	0	1	P+V	NA	15	0	15	1
19	F	58	O	1	IV	T4 N3 M1	R	26	4	P+V	PR	1	1	15	1
20	M	65	S	1	IIIB	T4 N2 M0	L	26	2	P+V	PD	2	1	14	1
21	F	57	A	1	IV	T3 N1 M1	L	14	3	P+V	SD	6	1	7	1
22	M	76	S	1	IIIB	T2 N3 M0	R	0	1	P+V	NA	4	0	4	1
23	M	72	O	1	IIIA	T4 N0 M0	R	0	1	P+V	NA	6	0	6	1
24	M	71	S	1	IIIB	T4 N3 M0	R	0	1	P+V	NA	1	0	1	1
25	M	73	S	1	IIIA	T3 N2 M0	R	14	2	P+V	NA	4	0	4	1

Figure A. Global sensitivity analysis

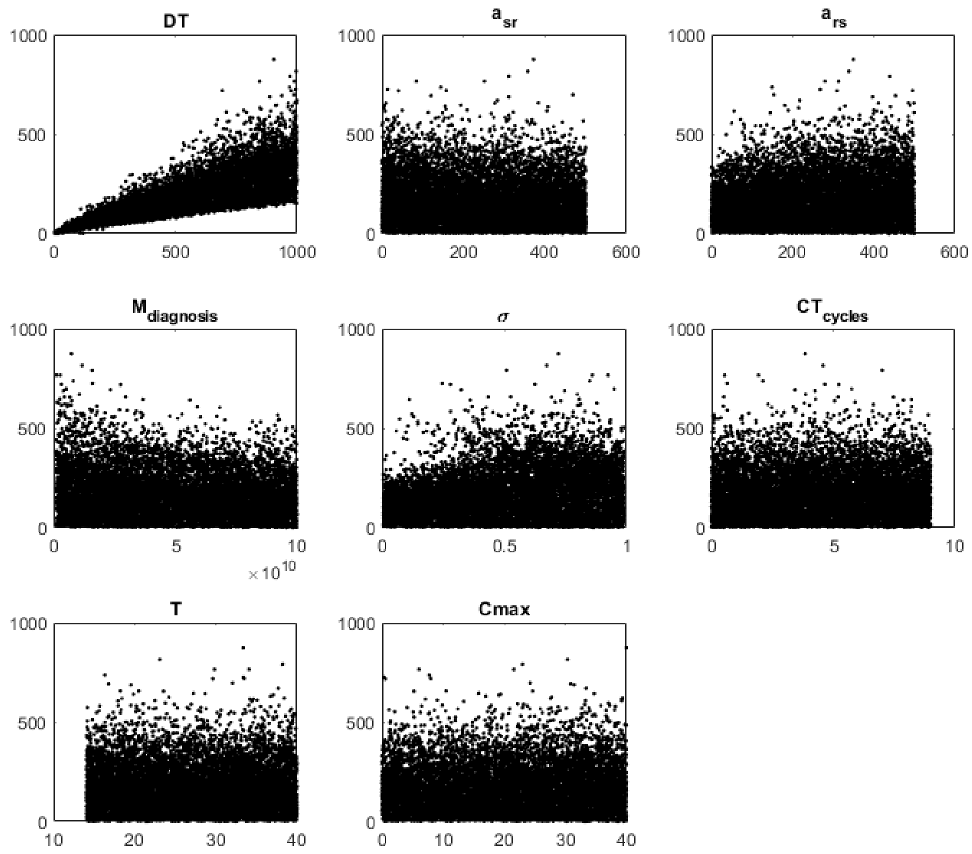

Figure A: Global sensitivity analysis. The plots show the dependence of each model parameter on overall survival. Each dot on the plot represent one parameter combination.

Figure B. Probability density function of (DT, σ)

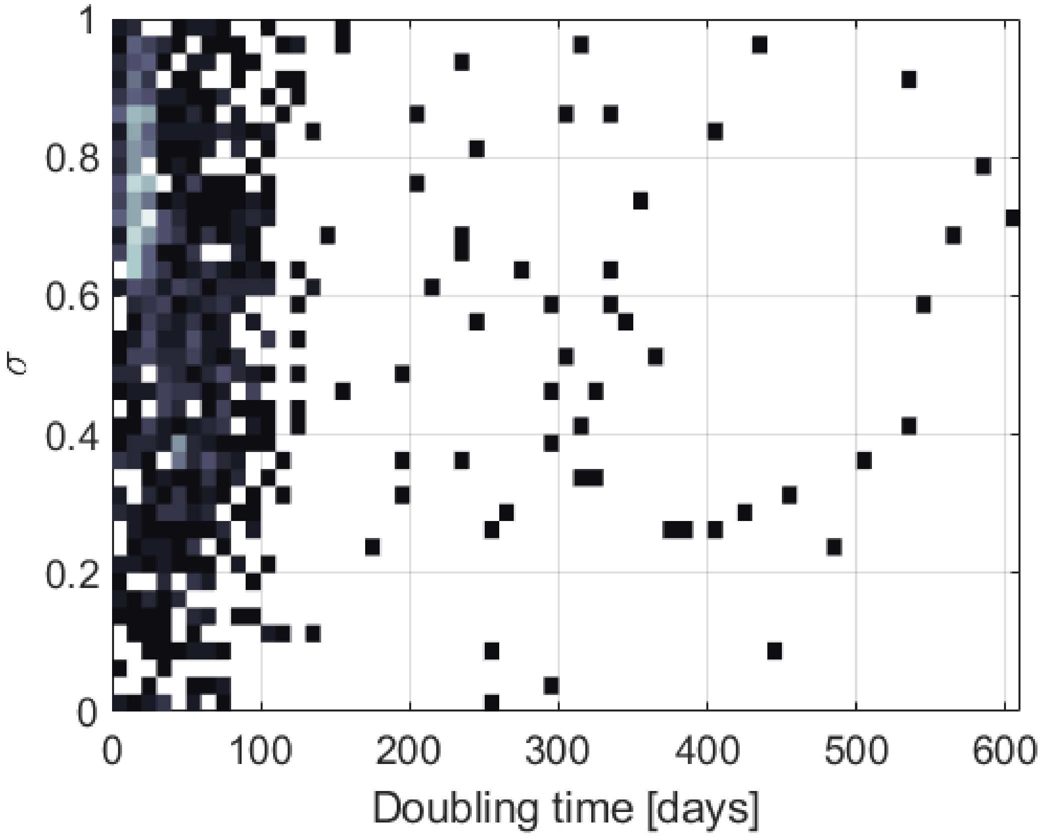

Figure B: The histogram shows the joint probability density function of two parameters DT and σ.

Figure C. Probability density function of (CT_{cycle}, T)

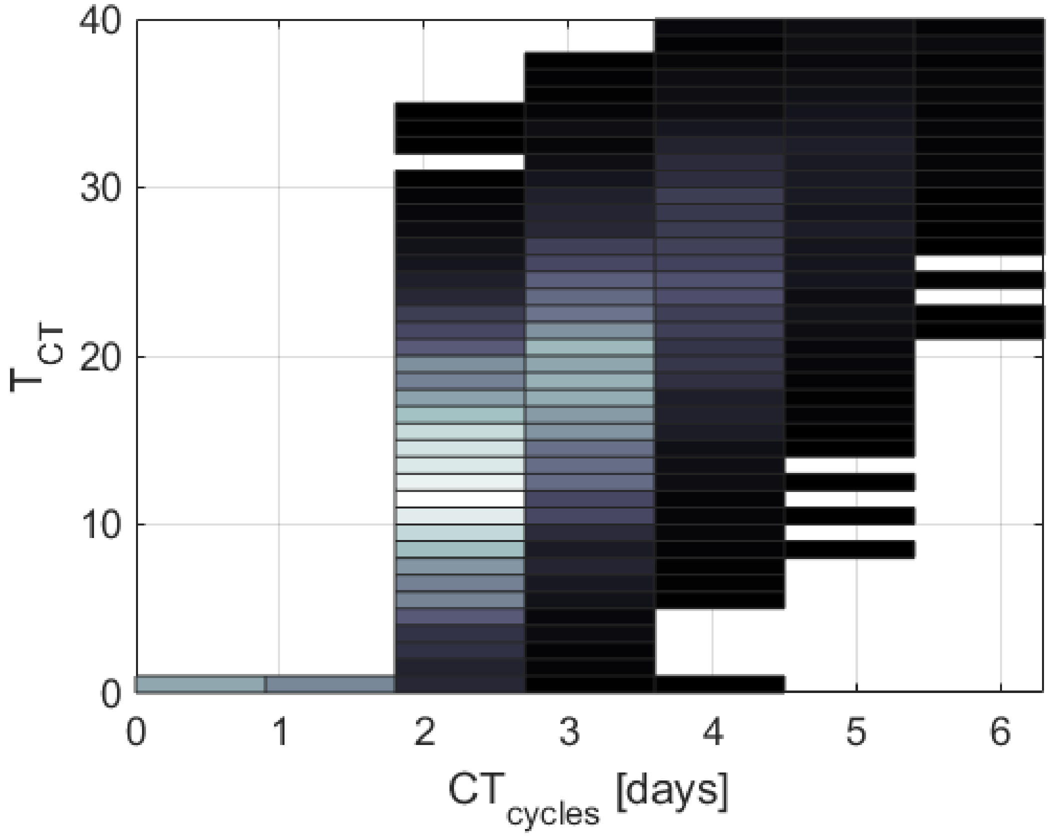

Figure C: The histogram shows the joint probability density function of two parameters CT_cycle and T.

Figure D. Probability density function of (DT, σ) for each clinical patient

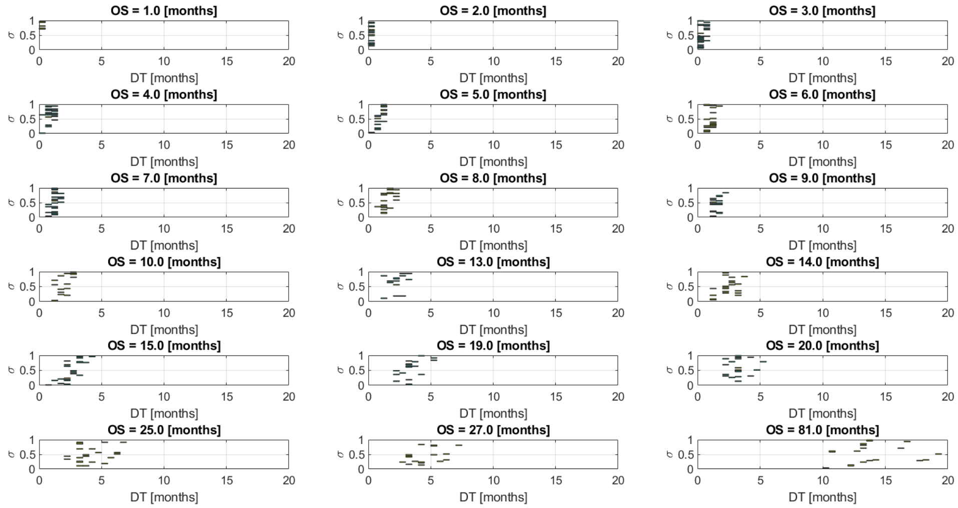

Figure D: The histogram shows the joint probability density function of two parameters DT and σ for each overall survival value observed in clinical cohort.
